# Adolescents’ risky sexual behaviours and practices: Implications for sexuality education implementation in Zambia

**DOI:** 10.4102/phcfm.v16i1.4476

**Published:** 2024-07-26

**Authors:** Bright Mukanga, Siyabonga B. Dlamini, Ngoy Mwanabute, Myra Taylor

**Affiliations:** 1 Discipline of Public Health Medicine, School of Nursing and Public Health, University of KwaZulu-Natal, Durban, South Africa; 2 Department of Public Health, School of Medicine, Copperbelt University, Ndola, Zambia; 3 Cancer and Infectious Diseases Epidemiology Research Unit, College of Health Sciences, University of KwaZulu-Natal, Durban, South Africa; 4 Department of Mathematics, School of Mathematics and Natural Sciences, Copperbelt University, Kitwe, Zambia

**Keywords:** risky sexual behaviour, comprehensive sexuality education, adolescents, Kitwe, Zambia

## Abstract

**Background:**

Adolescents’ risky sexual behaviours (RSB) are detrimental to their sexual and reproductive health (SRH) well-being and present a serious public health threat, particularly in low- and middle-income countries (LMICs).

**Aim:**

This study aims to assess RSB among Grade 12 school-going adolescents after exposure to comprehensive sexuality education (CSE).

**Setting:**

This study was conducted in Kitwe district, Zambia.

**Methods:**

This cross-sectional study included 807 Grade 12 pupils at 13 selected secondary schools. Data were collected using a structured questionnaire. Proportionate probability sampling involving 13 schools was employed. Risky sexual behaviours binary outcome variables were based on transactional sex, sex while drunk, multiple sexual partners, age-disparate sexual relationships, and condomless sex. We conducted univariate and bivariate analyses to summarise sociodemographic factors and fitted binary and multivariable logistic regression models.

**Results:**

The prevalence of RSB was 40.4%. Drinking alcohol (adjusted odds ratio [AOR] = 20.825; 95% CI [6.7–64.489]); ever had sex (AOR = 9.024; 95% CI [1.953–41.704]); school location (AOR = 6.50; 95% CI [1.61–26.24]); living with mother only (AOR = 4.820; 95% CI [1.328–17.493]); sex (male) (AOR = 2.632; 95% CI [1.469–4.713]), watching pornography (AOR = 1.745; 95% CI [1008–3.021]); religion (AOR = 0.472; 95% CI [0.250–0.891]) and attending religious functions (AOR = 0.317; 95% CI [0.118–0.848]) were significantly associated with RSB. Of the sexually active pupils, 221 (67.7%), 64 (19.6%) and 41 (12.5%) were in the low, medium and high-risk categories, respectively.

**Conclusion:**

Close to half of the respondents engaged in RSB. This is a significant number that needs intervention. The CSE programme needs to be linked with structural programmes that address the social drivers of RSB among adolescents.

**Contribution:**

The study provides a backdrop for evaluating current CSE strategies in LMICs.

## Introduction

Globally, the human immunodeficiency virus/acquired immune deficiency syndrome (HIV/AIDS) epidemic continues to be a public health threat, especially in the sub-Saharan African (SSA) region.^1^ Despite long decades of global efforts to reduce the mortalities and morbidities associated with HIV, 39.0 million people were living with HIV in 2022 globally, and 65% of people living with HIV reside in SSA.^2^ A significant rise in the number of new HIV infections among adolescents and young people (AYP) is a global health concern. In particular, about 27% of the 1.3 m new HIV infections worldwide in 2022 occurred in people between ages 15 and 24 years.^3^ Six out of seven new HIV infections occur among adolescents aged 15–19 with young women aged 15–24 twice as likely to be living with HIV compared to their male counterparts in SSA.^4^ Adolescents are disproportionately affected by HIV because of biological, socioeconomic and cultural-related factors.^5^

The World Health Organization defines an adolescent as a person aged 10–19 years, and this overlaps with that of young people who are categorised between 10 and 24 years.^6^ The adolescence period is marked by psychosocial, physiological and cognitive changes that can arouse sexual curiosity and desire to experiment with risky sexual behaviours (RSB).^7^ This vulnerability is compounded by the broader social and economic conditions which include intimate partner violence and lack of access to basic needs such as education, housing and food security.^5^ Gender inequalities and harmful cultural norms also expose adolescents, especially girls to a high risk of HIV transmission.^8^ Alongside biomedical interventions, structural interventions that address multilevel risk factors have been employed to reduce the HIV incidence among adolescents.^9^ These may include interventions such as anti-poverty cash transfers, caregiver support programmes and gender transformative participatory approaches.^5^

Multiple studies have defined RSB as any sexual activity that increases the chance of acquiring sexually transmitted infections (STIs) and unwanted pregnancies.^10,11^ These include activities such as early sexual debut, condomless sex, consuming alcohol before sex, multiple sexual partners, transactional sexual relationships and age-disparate sex.^12,13,14,15,16^ Chawla and Sarka^17^ have defined RSB as any sexual activities that expose the person to a high risk of contracting HIV/AIDS. Risky sexual behaviours among adolescents are driven by structural factors such as low income and limited access to financial resources, lack of awareness about RSH issues and harmful traditional practices.^18^ Other factors associated with RSB include being sexually abused, watching pornography, poor supervision by parents, peer pressure and drinking alcohol.^19^ However, the definition of RSB remains contested in extant literature owing to variations in culture, gender, age and the threshold to which the concept may be applied.^17^

School-based comprehensive sexuality education (CSE) is an effective strategy to enhance adolescents’ access to sexual and reproductive health (SRH) information to enable them to make informed decisions regarding their sexuality.^20^ Comprehensive sexuality education has been defined as an ‘age-appropriate, culturally relevant approach to teaching about sexuality and relationships by providing scientific, accurate, realistic and non-judgmental information’.^21^ Some of the benefits of CSE implementation include delaying sexual debut, reducing the number of sexual partners and unwanted pregnancies and increasing safer sexual practices.^22^ Comprehensive sexuality education is also essential in demystifying wrong perceptions and myths about adolescent sexuality.^23^ However, a profound criticism of the CSE programme is that it does not address structural and contextual factors influencing sexual behaviours among adolescents.^24^

Zambia is one of the countries in SSA with a high burden of HIV and with a national HIV prevalence of 11.0% reported in 2021.^25^ The HIV epidemic in Zambia is mainly driven by unprotected heterosexual sex and inconsistent condom use.^26^ Zulu et al.^22^ reported that 30% of adolescent girls aged 15–19 had begun childbearing in 2019. Contextual factors such as sexual violence, limited sexual decision making and transactional sex have been found to increase the HIV risk, especially among adolescent girls and young women (AGYW).^27^ To address RSB and limited access to SRH knowledge among adolescents,^22^ the government of Zambia introduced the new 2014 CSE framework in all secondary and primary schools and targeted adolescents enrolled in grades 5–12.^23^ The revised CSE framework contains six thematic areas which include: values, attitudes and skills; culture, society and human rights; human development; and sexual behaviours and is centred on delaying adolescents’ sexual debut, reducing the number of sexual partners and increasing safer sexual practices.^22,28^

Although the Zambian CSE framework stresses emphasis on the right-based approach to teaching about sexuality, most teachers emphasise the abstinence-only approach because of inherent social-cultural barriers.^22^ In addition, Yang et al.^29^ report that in 2019, 72% of young people had engaged in RSB in Zambia. In a nationally representative survey among female adolescents aged 15–19 years old, Sserwanja et al.^6^ reported a 71% prevalence of RSB among adolescents, which included intercourse before age 16, condomless sex, engaging in transactional sex, sex while drunk and multiple sexual partners. Invariably, teachers skip sensitive topics such as pornography and homosexuality which conflict with their religious and cultural beliefs.^22,23^ To the best of our knowledge, this is the first study to have assessed RSB among school-going adolescents after cumulative exposure to the CSE programme in Zambia. Findings from this study have implications for sexuality education implementation in Zambia.

## Research methods and design

### Study setting

This was an institutional cross-sectional study that was carried out at 13 selected secondary schools (nine public and four private) in the Kitwe district of the Copperbelt Province. Six schools were urban, one was rural and six were semi-urban. The Copperbelt province has a population of 2.669635^25^ and the third-highest HIV prevalence of 14.2%.^30^ Kitwe district has 16 public secondary schools and 17 private secondary schools. Social and structural vulnerabilities exist and contribute to the increase in HIV acquisition among school-going adolescents.^31^

### Sampling and target population

We targeted Grade 12 pupils from randomly selected secondary schools. Proportionate probability sampling stratified as public and private was employed. We employed a two-stage stratified cluster sampling to select the participants. In the first stage, we selected schools stratified as public or private proportionate to the size of each school. The second stage involved the random selection of Grade 12 classes; all pupils from the selected classes were included in the study.

### Inclusion and exclusion criteria

We included all the selected Grade 12 pupils who had been learning CSE from grades 8 up to grade 12 and those who were available on the day of data collection. We excluded pupils from grades 8 to 11 and those who were mentally disabled. Mentally disabled pupils were identified with the help of guidance teachers in each school.

### Study design

An institutional-based cross-sectional design was employed to assess the prevalence of RSB and associated factors among grade 12 school going adolescents after cumulative exposure to CSE. This design was essential in highlighting associations between predictor variables and response variables. The study was conducted from October 2022 to April 2023.

### Sample size estimation

We employed the following sample size parameters in the following equation to calculate the sample sizes for the pupils:


$$\begin{array}{l} n=\frac{Z^{2}\times P(1-P)}{d^{2}}\\ n\times \text{Deff}=n_{\text{total}} \end{array}$$ [Eqn 1]

where:

*n* is the sample size of an infinite population;

*Z* is the *Z* table statistic at the 95% confidence level;

*P* is the expected proportion of the target population with correct knowledge set at 50%;

*d* is the precision, set at 5%;

Deff is the design effect for two-stage cluster sampling at 2? (depends on average responses/cluster).

The calculated sample size was 385 × 2 = 770 pupils and the 10% expected nonresponse = 847. A total of 847 pupils were sampled.

### Data collection

Data were collected using a validated and standardised pre-tested structured anonymous questionnaire that was adapted from the previous literature.^32^ The questionnaire had sections on demographic information, eight questions that described sexual experiences, eight questions on HIV knowledge, gender norms, body image perception and RSB-related questions. The questionnaire was originally written in English and was then translated to the local language (Bemba) by a local language translator. Data collection was done with the help of data collectors who were earlier trained in quantitative research. We pretested the questionnaire in four schools that were not part of the schools selected to participate in the study to identify any flaws in the questionnaire. Furthermore, we measured the internal reliability between the items on the questionnaire using STATA version 18 and obtained the scale reliability coefficient (Cronbach’s alpha) of 0.8.

### Measurements and operational definitions

The outcome variable in this study was RSB which was measured using a composite RSB that included five outcome indicators such as *Transactional sex* (as a receipt of money, drinks, clothes, cell phones, better marks at school and school fees); *Multiple sexual partners* (as having more than one sexual partner); *Sex while drunk* (as having sexual intercourse while under the influence of alcohol); *Age disparate sexual relationship* (as having a sexual partner at least 10 years older) and *Condomless sex* (as not using a condom for the duration for sex in last sex).^5^ For questions with binary outcomes, YES was coded 1 while NO was coded 2. The total possible score was 5 points, if respondents answered Yes to any of these outcome variables, it was defined as RSB.^6,17^ We used the composite total risk score of 5 to measure no-risk (0), low-risk (1), medium-risk (2) and high-risk behaviours (3–5). Participants who reported not having had sex were categorised as not having any sexual risk outcome.

### Data analysis

Data were cleaned using Microsoft Excel (Microsoft, Redmond, WA, USA) and exported into STATA software version 2018 (Stata Corporation, College Station, TX, USA). We conducted univariate and bi-variate analyses to summarise sociodemographic factors. To select the independent variables to be included in the logistic model, the Chi-square of independence was performed on data to assess any association or relationship between the response variables and the potential predictor variables using the backward stepwise regression method. Variables with *p*-value ≤ 0.05 were fitted into a multivariable logistic regression model to assess predictors of RSB while controlling for potential confounders. The selection of covariates was also based on previous studies.^5,33,34,35^ The logistic regression model suitability was examined with the Hosmer–Lemeshow goodness-of-fit test and the goodness-of-fit measure (Nagelkerke’s *R*^2^) (see Table 1). We used adjusted and unadjusted odds ratios with 95% confidence intervals to determine associations between primary outcome variables and predictor variables. We aggregated the composite score of the outcome variables by adding the values of the individual response variables; the multinomial multiple logistic regression method was used. As this aggregate variable had six values ranging from 0 to 5, 0 was selected as the baseline category and five binary multiple logistic models were formed by comparing categories 1–4 with the baseline category. Choosing ‘no risk’ as the baseline category, the multinomial multiple logistic regression model comprised three binary logit functions to predict the log odds of low risk versus no risk, medium risk versus no risk and high risk versus no risk. Thus, the aggregate variables which had six values were reduced to four values 0 = no risk, 1= low risk, 2 =medium and 3–5 = high risk).

**TABLE 1:** Results for the likelihood ratio tests, the Hosmer and Lemeshow tests and Nagelkerke’s *R*^2^.

Response variable	LRT: G^2^ – value	*p*	HLT: χ^2^ – value	*p*	Nagelkerke’s *R*^2^	Rate of correct classification (%)
Age-disparate sexual relationships	60.925	0.000	3.812	0.874	0.113	76.3
Sex while drunk	101.914	0.000	5.365	0.718	0.456	96.9
Transactional sex	100.141	0.000	5.681	0.683	0.348	94.9
Multiple sexual partners	102.534	0.000	4.821	0.777	0.272	91.8
Condomless sex	113.781	0.000	2.848	0.944	0.314	72.2

LRT, likelihood ratio tests; HLT, the Hosmer and Lemeshow tests; G2; test of goodness-of-fit; *P, p*-value; χ^2^, chi-square; R^2^, coefficient of determination.

### Conceptual framework

**FIGURE 1:**
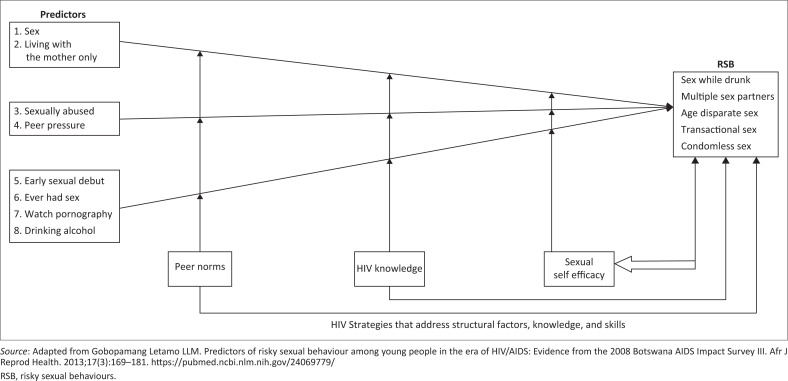
Risk and protective model for predicting risky sexual behaviour.

We employed a theory-driven theoretical model that highlights the possible risks and protective factors among school-going adolescents. The model posits that the influence of risk factors on risk behaviours can be mediated by protective factors including strategies that address structural factors that increase the HIV risk. Using this framework, we hypothesised that adolescents who reported having had sex before, watched pornography, had an early sexual debut, were sexually abused, were pressured to have sex by their peers, had sex while drunk and were boys would report engaging in any of the outcome variables (Figure 1). As such, young people who have high knowledge of HIV, high sexual self-efficacy and positive peer norms (believe their peers practice safer sexual practices) are likely to report fewer RSB.^5,36^

### Ethical consideration

Approval for the study was granted by Tropical Diseases Research Centre in Ndola, Zambia (IRB Registration Number: 00002911 and FWA Number: 00003729). Permission was granted by the National Health Research Authority (NHRA), Zambia (Ref No: NHRA000001/01/06/2022) and the Biomedical Research Ethics Committee of the University of KwaZulu-Natal in Durban, South Africa (Approval number: BREC/00004141/2022). Written informed consent was obtained from all participants before data collection. We followed the standard procedures of informed consent, which included anonymity and confidentiality. Before the actual data collection, letters of support were obtained from the District Education office and all the participating schools. For adolescents under the age of 18 years, we obtained consent from their parents and guardians to participate in the study. Participants were informed about the purpose, benefits and possible harms of the study. We anonymised all data records and ensured privacy and confidentiality.

## Results

### Summary of descriptive characteristics

In total, 807 Grade 12 school-going adolescents responded to this study (40 questionnaires were removed because of missing information). There were 332 (41.1%) boys and 475 (58.8%) girls, of whom 386(47.8%) were from the peri-urban region, 94 (11.6%) from the rural and 327 (40.5%) from urban areas. The mean age for both girls and boys was 17.6, range (17.6–17.8). Of the 326 (40.4%) who ever had sex, 157 (48.2%) were girls, 169 (51.84%) were boys and 481 (59.6%) had not experienced sex. Of the sexually active pupils (326), 68 (20.7%) had multiple sexual relationships, 30 (9.2%) had sex while drunk (boys, 16 [4.9%] and girls, 14 [4.3%]), 161 (48.7%) did not use a condom at the last sex, 198 (60.7%) were in age-disparate sexual relationships and 42 (12.8%) had engaged in transactional sexual relationships. Of these pupils, 54 (16.5%) had sex before the age of 16, with a mean age at sex debut of 16.5 (see Table 2).

**TABLE 2:** Socio-demographic characteristics by sex in Kitwe district (*N* = 807).

Characteristic	Male (*n* = 332)		Female (*n* = 475)		Overall (*N* = 807)		*p*
	*n*	%	*n*	%	*n*	%	
**School admission**							
Boys and girls	332	43.6	430	56.4	762	94.4	< 0.001
Girls only	-	-	45	100	45	5.6	-
**School location**							
Peri-urban	183	47.4	203	52.5	386	47.8	< 0.001
Rural	44	46.8	50	53.2	94	11.6	-
Urban	105	32.1	222	67.9	327	40.5	-
**Residential area**							
Peri-urban	183	55.1	203	42.7	386	47.8	< 0.001
Rural	44	13.25	50	10.53	94	11.6	-
Urban	105	31.63	222	46.74	327	40.5	-
**School sponsorship**							
Government aided	289	42.4	393	57.6	682	84.5	0.096
Private owned	43	34.4	82	65.6	125	15.5	-
**Age at last birthday (years)**							
15–16	31	23.0	104	77.0	135	16.7	< 0.001
17–18	197	39.3	304	60.7	501	62.1	-
≥ 19	104	60.8	67	39.2	171	21.2	-
Likes attending religious functions	186	36.3	326	63.6	512	100	< 0.001
Has school uniform	253	43.4	330	56.6	583	100	0.036
Had been sexually abused before	99	37.6	164	62.4	263	100	0.160
Physically abused by a sexual partner	38	45.78	45	54.22	83	100	0.364
Could convince a partner to use a condom	191	42.6	257	57.4	448	100	0.335
Ever had sex	169	51.8	157	48.1	326	100	< 0.001
Drinks alcohol	69	49.6	70	50.4	139	100	0.025
Knows his/her HIV status	203	42.4	276	57.6	479	100	0.387
Knows partner’s HIV status	82	41.2	117	58.8	199	100	0.983
Lives with their mother	258	41.0	371	58.9	629	100	0.894
Feels pressured to have sex	246	46.8	279	53.1	282	100	< 0.001
Watched pornography	145	44.8	178	55.1	323	100	0.077
Had sex while drunk	16	53.3	14	46.7	28	100	0.382
Transactional sex	20	47.6	22	52.4	42	100	0.381
Condomless last sex	92	57.14	69	42.9	161	100	< 0.001
In an age-disparate sexual relationship	82	41.4	116	58.6	198	100	0.928
Had multiple sexual partners.	45	66.2	23	33.8	68	100	< 0.001
**Age at sex debut (years)**							
≤ 16	18	33.3	36	66.7	54	16.4	< 0.001
≥ 16	154	56.0	121	44.0	275	83.6	-

Note: Mean age in years: 17.6–17.8 years (17.6); 15–16 years (15.9); 17–18 years (17.5); ≥ 19 years (19.7); Boys (18.1); Girls (17.4).

### Results from the multinomial multiple logistic regression model

#### Risky sexual behaviour assessment

Results from the aggregated response score indicated that of the 326 sexually active pupils, 221 (67.7%) were in the low-risk category, 64 (19.6%) were in the medium-risk category and 41 (12.57%) were in the high-risk category. The prevalence of RSB was 40.4%. When comparing ‘low-risk’ to ‘no-risk’ school location (*p* = 0.003; OR = 3.42), ever had sex (*p*-value 0.026; OR=2.07) and insisting on condom use (*p* = 0.000; OR = 6.89) were statistically significant. For ‘medium risk’ compared to ‘no risk’, significant predictor variables were school location (*p* = 0.036, OR = 0.391), ever had sex (*p* = 0.010, OR = 0.275) and drinking alcohol (*p* = 0.019, OR = 2.516). For ‘high risk’ compared to ‘no risk’, drinking alcohol (*p* < 0.001, OR = 6.044) and genitals fondled (*p* = 0.026, OR = 4.504) were found to have significant effects on the response variable. Compared to urban schools, pupils in rural schools were less likely to be in the “low risk” and “medium risk” categories when other predictor variables were kept constant. For the predictor insisting on condom use, learners who could convince their partners to use condoms when having sex were less likely to fall into any of the three risk categories. When comparing high-risk to no-risk, it was observed that pupils who ever had sexual intercourse were more likely to fall in the high-risk category. For pupils who were sexually abused, the odds of falling into the ‘low risk’, ‘medium risk’ or ‘high risk’ categories were respectively 1.881, 3.244 and 2.92 times higher than those who were not sexually abused.

### Results from multivariable regressions

#### Associations with risky sexual behaviours

In multivariable logistic regression analysis, school admission, school location, sex, like attending religious functions, sexually abused before, physically abused before, having been fondled before, insisting on condom use, ever had sex, drinking alcohol, school uniform, living with a mother, knowing HIV status, knowing the partner’s HIV status, pressured to have sex, age and watching pornography on the smartphone were significantly associated with RSB.

#### Factors associated with sex while drunk with alcohol

Keeping urban as a baseline category, the odds of having sex while drunk for pupils in peri-urban schools were 3.589 times higher than those in urban schools (OR = 3.585; 95% CI [1.054–12.195]). The odds of having sex while drunk for learners in rural schools were 6.505 times higher than those in urban schools (OR = 6.505; 95% CI [1.612–26.248]). For the predictor variable drinking alcohol, the odds of having sex while drunk for those who were drinking alcohol were 20 times higher than those who were not drinking alcohol (OR = 20.85; 95% CI [6.725–64.489]) (see Table 3).

**TABLE 3:** Bivariate and multivariable logistic regressions for factors associated with sex while drunk: Model 1.

Predictor variable	uOR	*p*	95% CI		aOR	*p*	95% CI	
			Lower	Upper			Lower	Upper
School location 1: Peri Urb	2.604	0.067	0.936	7.243	3.585	0.041	1.054	12.195
School location 2: Rural	5.991	0.002	1.911	18.776	6.505	0.009	1.612	26.248
School location 3: Urban	Ref	-	-	-	Ref	-	-	-
Likes attending religious services	0.260	0.001	0.116	0.582	0.317	0.022	0.118	0.848
Sexually abused before	2.471	0.019	1.158	5.271	0.748	0.643	0.219	2.554
Physically abused before	3.083	0.013	1.269	7.490	1.696	0.342	0.570	5.045
Ever had sex	20.757	< 0.001	4.891	88.086	9.024	0.005	1.953	41.704
Ever drunk alcohol	34.643	< 0.001	11.802	101.694	20.825	< 0.001	6.725	64.489
Had genitals touched without consent	3.144	0.003	1.464	6.749	1.878	0.312	0.554	6.371
Ever watched pornography	2.389	0.027	1.104	5.169	1.266	0.618	0.501	3.201
Constant	−8.356	39.438	-	-	-	< 0.001	-	-

uOR, unadjusted odds ratio; aOR, adjusted odds ratio; Cl, confidence interval; *P, p*-value.

#### Factors associated with transactional sex

The predictor variables ever had sex before (OR = 6.29; 95% CI [2.260–17.532]), drinking alcohol (OR = 2.63; 95% CI [1.243–5.558]) and living with a mother (OR = 4.82; 95% CI [1.328–17.493]) were significantly associated with being in a transactional sexual relationship. The odds of having sex in exchange for money or gifts for pupils who lived with their mothers only were 4.82 times higher than those who lived with both mother and father. The odds of having received money or gifts in exchange for sex for pupils who ever had sex were 6.29 times higher than those who did not have sex. For the predictor, drinking alcohol, the odds of having sex in exchange for money or gifts for pupils who were drinking alcohol were 2.63 times higher than those who were not drinking alcohol (see Table 4).

**TABLE 4:** Bivariate and multivariable logistic regressions for factors associated with transactional sex: Model 2.

Predictor variable	uOR	*p*	95%CI		aOR	*p*	95% CI	
			Lower	Upper			Lower	Upper
School location Urban 3	Ref	-	-	-	Ref	-	-	-
School location: Urban 1	2.247	0.026	1.101	4.587	1.753	0.179	0.773	3.975
School location: Rural 2	0.947	0.935	0.259	3.467	0.449	0.264	0.110	1.828
Likes attending religious functions	0.504	0.031	0.270	0.941	0.545	0.108	0.260	1.143
Lives with mother at home	3.866	0.026	1.177	12.629	4.820	0.017	1.328	17.493
Sexually abused before	6.475	< 0.001	3.199	13.102	2.685	0.062	0.950	7.589
Physically abused before	2.962	0.005	1.399	6.271	1.395	0.447	0.592	3.288
Ever had sex	12.188	< 0.001	4.736	31.366	6.295	< 0.001	2.260	17.532
Ever drunk alcohol	4.935	< 0.001	2.612	9.325	2.628	0.011	1.243	5.558
Knows partner’s HIV status	3.654	< 0.001	1.949	6.850	1.932	0.067	0.956	3.907
Pressured to have sex	2.806	0.014	1.230	6.402	1.801	0.214	0.712	4.554
Had genitals fondled	5.562	< 0.001	2.838	10.898	1.606	0.354	0.590	4.371
Watched pornography	1.876	0.048	1.005	3.504	0.947	0.882	0.459	1.952
Age group 3: ≥ 19 years	Ref	-	-	-	Ref	0.192	-	-
Age group 1: 15–16 years	0.655	0.351	0.269	1.594	1.232	0.701	0.425	3.573
Age group 2: 17–18 years	0.410	0.013	0.203	0.826	0.564	0.160	0.254	1.253
Constant	−7.569	-	-	-	0.001	< 0.001	-	-

uOR, unadjusted odds ratio; aOR, adjusted odds ratio; Cl, confidence interval; *P, p*-value.

#### Factors associated with age-disparate sexual relationships

The response variable age-disparate sexual relationship was associated with predictor variables religion (OR = 0.472; 95% CI [0.250–0.891]), ever had sex (OR = 1.485; 95% CI [1.030–2.140]) and efficacy to insist on condom use (OR = 1.477; 95% CI [0.983–2.220]). Compared to pupils who were Christians, pupils with no religion were more likely to be in an age-disparate sexual relationship. For pupils who had sexual intercourse before, the odds of being in an age-disparate sexual relationship were 1.485 times higher than those who had not had sexual intercourse (see Table 5).

**TABLE 5:** Bivariate and multivariable logistic regressions for factors associated with age-disparate sexual relationships: Model 3.

Predictor variable	uOR	*p*	95%CI		aOR	*p*	95% CI	
			Lower	Upper			Lower	Upper
Religion (none)	Ref	-	-	-	Ref	-	-	-
Religion (Christian)	0.430	0.005	0.238	0.780	0.472	0.021	0.250	0.891
Religion (Islam)	2.537	0.178	0.655	9.829	2.913	0.143	0.697	12.181
Likes attending religious functions	0.576	0.001	0.416	0.798	0.721	0.069	0.507	1.026
Sexually abused before	1.874	< 0.001	1.346	2.609	1.091	0.736	0.658	1.809
Physically abused before	1.764	0.021	1.088	2.859	1.231	0.440	0.727	2.084
Ever had sex	2.094	< 0.001	1.513	2.898	1.485	0.034	1.030	2.140
Ever drunk alcohol	1.974	0.001	1.334	2.920	1.284	0.249	0.840	1.965
Efficacy to insist on condom use	2.048	< 0.001	1.433	2.927	1.477	0.060	0.983	2.220
Had genitals fondled	1.999	< 0.001	1.432	2.792	1.416	0.181	0.851	2.357
Ever watched pornography	1.541	0.009	1.115	2.130	1.229	0.241	0.871	1.734
Constant	-	10.460	-	-	0.326	0.001	-	-

uOR, unadjusted odds ratio; aOR, adjusted odds ratio; Cl, confidence interval; *P, p*-value.

#### Factors associated with multiple sexual partners

The predictor variables, sex (OR = 2.632; 95% CI [1.469–4.713]), ever had sex (OR = 7.139; 95% CI [3.349–15.220]) and watching pornography (OR = 1.745; 95% CI [1.008–3.021]) were significantly associated with having multiple sex partners. Compared to girls, boys were 2.63 more likely to have multiple sexual partners when other predictors were kept constant. Compared to pupils who never had sex, pupils who had sex before were 7.13 more likely to engage in multiple sexual relationships when other predictors were kept constant. Compared to those who never watched pornography, pupils who were watching pornography were 1.74 more likely to have multiple sexual partners after keeping other predictors constant (see Table 6).

**TABLE 6:** Bivariate and multivariable logistic regressions for factors associated with multiple sexual partners: Model 4.

Predictor variable	uOR	*p*	95%CI		aOR	*p*	95% CI	
			Lower	Upper			Lower	Upper
Sex: Boy	3.081	< 0.001	1.825	5.202	2.632	0.001	1.469	4.713
Sex: Girl (Ref)	Ref	-	-	-	-	-	-	-
Sexually abused	3.092	< 0.001	1.865	5.126	1.354	0.455	0.612	2.994
Physically abused	3.086	< 0.001	1.670	5.703	1.667	0.142	0.843	3.295
Ever had sex	11.589	< 0.001	11.589	23.743	7.139	< 0.001	3.349	15.220
Knows HIV status	1.853	0.028	1.070	3.211	1.642	0.129	0.865	3.118
Knows partner’s HIV status	2.663	< 0.001	1.601	4.428	1.545	0.156	0.847	2.817
Pressured to have sex	2.687	0.003	1.415	5.101	1.715	0.125	0.861	3.417
Had genitals fondled	2.987	< 0.001	1.806	4.940	1.414	0.391	0.640	3.124
Watched pornography	2.018	0.006	1.223	3.330	1.745	0.047	1.008	3.021
Age group 3: ≥ 19 years	Ref	-	-	-	Ref	0.299	-	-
Age group 1: 15–16years	0.438	0.043	0.196	0.976	1.146	0.766	0.467	2.809
Age group 2: 17–18 years	0.460	0.006	0.265	0.799	0.681	0.210	0.374	1.241
Constant	-	-	-	-	0.004	< 0.001	-	-

uOR, unadjusted odds ratio; aOR, adjusted odds ratio; Cl, confidence interval; *P, p*-value.

#### Factors associated with condomless last sex

When other predictor variables were kept constant, school location (OR = 2.726; 95% CI [1.304–5.697]), sex (OR = 1.649; 95% CI [1.030–2.642]), knowing partner HIV status (OR = 1.874; 95% CI [1.128–3.116]), ever had sex (OR = 7.692; 95% CI [3.623–16.332]) and efficacy to convince a partner to use condom (OR = 1.718; 95% CI [1.014–2.910]) were significantly associated with condomless last sex. Compared to pupils in urban schools, pupils in rural schools were 2.726 times more likely to use a condom in the last sex. Compared to girls, the odds of having used a condom in the last sex for boys were 1.649 times higher than for girls. For pupils who convinced their partners to use a condom, the odds of using a condom were 1.718 times higher than those who did not. Compared to pupils who never had sex, the odds of using condoms when having sex for pupils who ever had sexual intercourse were 7.692 times higher than those who never had sex. Pupils who knew their partner’s HIV status were 1.87 more likely to use condoms in the last sex compared to those who did not know their partner’s HIV status (see Table 7).

**TABLE 7:** Bivariate and multivariable logistic regressions for factors associated with condomless last sex: Model 5.

Predictor variable	uOR	*p*	95% CI		aOR	*p*	95% CI	
			Lower	Upper			Lower	Upper
School sponsorship	0.502	0.047	0.254	0.992	1.224	0.631	0.536	2.792
School admission	0.294	0.014	0.111	0.781	1.328	0.640	0.405	4.349
School location	-	-	-	-	-	0.023	-	-
School location: Peri-urban	0.560	0.011	0.358	0.876	1.247	0.426	0.724	2.150
School location: Rural	0.269	< 0.001	0.147	0.493	2.726	0.008	1.304	5.697
Sex	0.463	< 0.001	0.312	0.688	1.649	0.037	1.030	2.642
Efficacy to convince a partner to use a condom	0.348	< 0.001	0.222	0.547	1.718	0.044	1.014	2.910
Ever had sex	0.093	< 0.001	0.045	0.190	7.692	< 0.001	3.623	16.332
Knows HIV status (1)	0.591	0.012	0.392	0.890	1.213	0.454	0.732	2.008
Knows partners HIV status (1)	0.475	< 0.001	0.316	0.715	1.874	0.015	1.128	3.116
Could insist on using a condom	0.303	< 0.001	0.180	0.512	2.122	0.013	1.176	3.830
Age								
Age (1)	0.001	< 0.001	1.645	6.683	0.750	0.484	0.335	1.679
Age (2)	1.722	0.018	1.098	2.700	0.812	0.419	0.489	1.346
Constant	-	-	-	-	0.013	< 0.001	-	-

uOR, unadjusted odds ratio; aOR, adjusted odds ratio; Cl, confidence interval; *P, p*-value.

## Discussion

The study aimed to assess RSB among grade 12 school-going adolescents after cumulative exposure to the CSE programme. The study found that pupils who were boys or girls, older, ever had sex, watched pornography, lived with their mothers only, drank alcohol, were pressured to engage in sex, were living in rural and peri-urban areas, were physically and sexually abused before and were not religious were more likely to engage in RSB. In the current study, the prevalence of RSB was 40.4%. This finding is lower than findings reported elsewhere, where the prevalence of RSB was 80.2%, 69.5%, 72.2% and 71%, respectively.^6,29,34,37^ The observed difference in prevalence could be because of differences in social demographic variables in different contexts. These variations could be because of differences in sample size and the context in which the concept of RSB is applied.^34,38^

In the current study, 83 (10.3%) and 263 (32.6%) pupils reported having been physically and sexually abused, respectively. Although physical and sexual abuse were not associated with RSB at the multivariable level, at the multinomial level, pupils who were sexually abused were more likely to fall in the high RSB category. Conversely, other studies elsewhere have linked physical and sexual abuse to RSB at the multivariable level.^34,39,40^ Adolescents who are sexually and physically abused in childhood have increased odds of engaging in premature sexual debut, having multiple sex partners, impulse sexual behaviour and unprotected sex at an older age.^41^ Exposure to physical and sexual abuse during childhood can negatively impact the child’s development leading to cognitive, physiological and social dysfunction, which can result in limited life skills, making them vulnerable to subsequent sexual abuse and HIV risk. One of the unique aspects of this study was that 99 (29.8%) boys were sexually abused. While sexual abuse of girls has been widely studied, data on the sexual abuse of boys and associated factors are limited. This could be because of the underreporting of sexual abuse cases among boys because of fear of consequences and associated social stigma and discrimination against restricted sexual behaviour such as homosexuality and the desire to uphold self-reliance.^42,43,44^ As such, the current CSE programme should be gender-sensitive and address factors that contribute to forcible sexual abuse among boys and girls.

Multiple studies have analysed the relationship between adolescent RSB and alcohol use.^45,46,47,48^ A recent meta-analysis found that alcohol consumption was strongly associated with RSB such as early sexual initiation, inconsistent use of condoms and multiple sexual partners.^47^ In the current study, school location and drinking alcohol were statistically associated with having sex while drunk. However, the odds of having sex while drunk with alcohol were reduced for pupils who liked attending religious services. Alcohol use before sex is likely to increase young adolescent’s sexual risk through exposure to risky sexual partners.^49^ Another study revealed that the likelihood of engaging in unprotected sexual intercourse increased when pupils were in a drunken state^50^ Alcohol consumption before and during sexual intercourse can reduce sexual inhibition which may affect adolescents’ judgement regarding their sexual behaviour. However, though the current CSE programme stresses emphasis on values and attitudes towards sexual behaviour, it does not explicitly highlight the risk of having sex under the influence of alcohol. The CSE programme would need to focus more on highlighting the risk of HIV transmission from having sex while drunk with alcohol. Importantly, prevention and sensitisation programmes at an early stage, not only in schools but also in homes and communities need to be implemented.^47^

The link between transactional sex and HIV risk among adolescents is highlighted in extant literature.^5,51,52,53,54,55^ This is a public health concern because men with whom young women have sex may belong to networks of sexually connected individuals who are at high risk for HIV transmission.^56,57^ Additionally, adolescent girls are less likely to decide the timing and conditions for sex because of relationship power inequality.^53,58^ In the current study, transactional sex was associated with living with the mother at home and drinking alcohol. These findings corroborate findings from elsewhere.^59,60^ Data on transactional sex in extant literature have largely focussed on younger girls and older men.^61^ Interestingly, we found that 20 (6.1%) of the adolescents (boys) had traded sex for money with older women. Also, adolescents who were living with their mothers were more likely to engage in transactional sex. Pichon et al.^55^ relate this to the mother’s unidirectional form of communication about transactional sex which is mostly in the form of threats, restricting movement and friendship to prevent girls from having sex. Adolescent girls’ desire to have consumables that their mother’s limited income cannot afford can propel them to seek alternative sources of income. As such, family-level CSE programmes targeting parent–daughter communication which could focus on equitable power dynamics in transactional sex and reducing fear surrounding parent–daughter communication is recommended.^55^ Comprehensive sexuality education in schools linked with poverty cash transfer programmes to increase economic support to school-going adolescent girls and their guardians have been found to lower RSB.^5,61^ In light of these findings, the CSE policy framework should be reoriented to address structural factors such as economic vulnerability and gender inequality.^62,63^ However, not all sexual relationships that involve exchange are risky, as such the focus should be more on identifying the conditions and circumstances in which transactional sexual relationships impart risk.^63^

Age-disparate sexual relationships have been linked with a high risk of HIV acquisition among adolescents.^14,62,64^ The plausible explanation is that HIV prevalence among men increases steeply with age and age-disparate male partners are more likely to be HIV positive compared to their younger female partners.^65^ In the current study, the odds of engaging in age-disparate sex were reduced for pupils who were Christians while the odds of engaging in age-disparate sex were increased for pupils who ever had sex. These results are consistent with findings from elsewhere.^15,66^ Conversely, other studies have linked education level and multiple sexual partnerships to age-disparate sexual relationships.^67,68^ However, these covariates may vary between populations because of different proximate determinants such as social and biological factors.^62^ A combination of CSE and structural interventions is needed to mitigate drivers of age-disparate sexual relationships to reduce HIV risk among adolescents.^69^

Condomless sex is one of the common risky sexual practices among school-going adolescents, particularly in low- and middle-income countries (LMICs).^15^ In the current study, pupils from urban schools were less likely to use condoms in the last sex. Also, compared to boys, girls were less likely to use condoms in the last sex. In another study, not using a condom at the last sex was associated with being younger (≥ 16 years), early sexual debut and multiple sexual partners.^37^ Interestingly, in multinomial regression, we noted that pupils in rural schools compared to those in urban schools were more likely to be in the high RSB category. These findings align with those reported elsewhere.^69,70^ While consistent condom use has been known to reduce HIV risk, teachers rarely emphasise condom use during CSE lessons because of conservative cultural beliefs that forbid sex before marriage among adolescents. As such, comprehensive teacher training and engagement during the development and implementation of CSE could help reconcile the teacher’s personal religious and cultural beliefs and the content of CSE.^71^

The relationship between pornography consumption and RSB has been established in the literature.^18,72^ However, this relationship remains contested as the risk depends on the frequency of viewing pornography.^73^ In the current study, pupils who were watching pornography were more likely to have multiple sex partnerships. Similar findings have been reported elsewhere.^74^ The plausible explanation for this observed relation is that the impulsive nature of pornographic materials can lead to erotic sex simulation and risky sexual practices.^18^ However, in most LMICs, topics such as pornography are never emphasised during CSE lessons because of inherent cultural and religious barriers.^22^ There is a need for increased dialogue with pupils and mutual engagement through strategic pupil-to-pupil communication and discussions during CSE lessons, where the effects of pornography consumption can be discussed.^75^ Parental influence could also be key in reinforcing positive sexual behaviours among adolescents. Specifically, through the provision of rules about viewing pornography during the adolescence period by instilling the beliefs in adolescents that disapprove pornography consumption.^75^

While multiple studies have highlighted the HIV risk of forcible sexual abuse and transactional sex among adolescent girls, future research is needed to understand the magnitude of sexual abuse and transactional sex and HIV risk among boys in schools. The study findings should be understood in light of the following limitations, because of the cross-section nature of the data, the results are limited to associations between variables and do not establish causal links. However, the large sample size enhanced the power of the study and sampling from a large geographical region ensured a representative sample. Social desirability bias could not be ruled out considering the sensitive topic and stigma surrounding the topic of sexuality among adolescents. We minimised this bias through anonymised questionnaires and participants were encouraged to skip questions they were not comfortable with.

## Conclusion

Close to half of the pupils had experienced some form of RSB. The prevalence of RSB among school-going adolescents was 40.4%. Comprehensive sexuality education linked with economic support programmes is needed to address structural drivers of HIV such as economic vulnerability and gender inequality, especially among girls. There is a need for CSE programmes to stress emphasis on gender-driven HIV risks such as forcible sexual abuse, sex under the influence of alcohol, age-disparate sex and condomless sex.

## References

[1] Moyo E, Moyo P, Murewanhema G, Mhango M, Chitungo I, Dzinamarira T. Key populations and sub-Saharan Africa’s HIV response. Front Public Health. 2023;16(11):1079990. 10.3389/fpubh.2023.1079990 PMC10229049 37261232

[2] Van Schalkwyk C, Mahy M, Johnson LF, Imai-Eaton JW. Updated data and methods for the 2023 UNAIDS HIV estimates. J Acquir Immune Defic Syndr. 2024;1(95):e1–e4. 10.1097/QAI.0000000000003344 PMC10769173 38180734

[3] Dzinamarira T, Moyo E. Adolescents and young people in sub-Saharan Africa: Overcoming challenges and seizing opportunities to achieve HIV epidemic control. Front Public Health. 2024;12:1321068. 10.3389/fpubh.2024.1321068 38566795 PMC10985137

[4] Murewanhema G, Musuka G, Moyo P, Dzinamarira T. HIV and adolescent girls and young women in sub-Saharan Africa: A call for expedited action to reduce new infections. IJID Reg. 2022;5(2772–7076):30–32. 10.1016/j.ijregi.2022.08.009 36147901 PMC9485902

[5] Rudgard WE, Saminathen MG, Orkin M, Banougnin BH, Shenderovich Y, Toska E. Protective factors for adolescent sexual risk behaviours and experiences linked to HIV infection in South Africa: A three-wave longitudinal analysis of caregiving, education, food security, and social protection. BMC Public Health. 2023;23(1):1452. 10.1186/s12889-023-16373-5 37516833 PMC10386676

[6] Sserwanja Q, Mwamba D, Poon P, Kim JH. Prevalence and factors associated with risky sexual behaviors Among sexually active female adolescents in Zambia. Arch Sex Behav. 2023;52(1):205–215. 10.1007/S10508-022-02385-6 36036870

[7] Zgambo M, Kalembo FW, Mbakaya BC. Risky behaviours and their correlates among adolescents living with HIV in sub-Saharan Africa: A systematic review. Reprod Health. 2018;15(1):180. 10.1186/S12978-018-0614-4 30355344 PMC6201550

[8] Baten J, De Haas M, Kempter E M zu SF. Educational gender inequality in sub-Saharan Africa: A long-term perspective. Popul Dev Rev. 2021;47(3):813–849. 10.1111/padr.12430

[9] Zuma T, Seeley J, Hlongwane S, et al. A socio-ecological approach to understanding experiences and perceptions of a multilevel HIV prevention intervention: The determined, resilient, empowered, AIDS-free, mentored, and safe (DREAMS) partnership in uMkhanyakude, KwaZulu-Natal, South Africa. Qual Res Health. 2022;2(100138):2667–3215. 10.1016/j.ssmqr.2022.100138

[10] Ndagijimana E, Biracyaza E, Nzayirambaho M. Risky sexual behaviors and their associated factors within high school students from Collège Saint André in Kigali, Rwanda: An institution-based cross-sectional study. Front Reprod Health. 2023;5:1029465. 10.3389/FRPH.2023.1029465 36936133 PMC10020213

[11] Kisaakye P, Bukuluki P, Wandiembe SP, et al. How self-efficacy and agency influence risky sexual behavior among adolescents in Northern Uganda. Adolescents. 2023;3(3):404–415. 10.3390/ADOLESCENTS3030028

[12] Agarwal N, Brar KB, Kumar S, Rajoa OS, Chahal A. Sexual behaviour and practices among adolescents and young people: Study and results from a tertiary care centre of north India. Int J Community Med Public Health. 2021;8(6):2937–2941. 10.18203/2394-6040.ijcmph20211997

[13] Osuala E, Ogbu B, Udi O, Osuala E, Ogbu B, Udi O. Risky Sexual behaviour among students of tertiary institutions in South-South, Nigeria: A qualitative study. Health (Irvine Calif). 2020;12(9):1095–1104. 10.4236/HEALTH.2020.129080

[14] Mabaso M, Maseko G, Sewpaul R, et al. Trends and correlates of HIV prevalence among adolescents in South Africa: Evidence from the 2008, 2012 and 2017 South African National HIV prevalence, incidence and behaviour surveys. AIDS Res Ther. 2021;18(1):1–8. 10.1186/S12981-021-00422-3/TABLES/4 34906170 PMC8670218

[15] Tekletsadik EA, Ayisa AA, Mekonen EG, Workneh BS, Ali MS. Determinants of risky sexual behaviour among undergraduate students at the University of Gondar, Northwest Ethiopia. Epidemiol Infect. 2022;150:e2. 10.1017/S0950268821002661 PMC8753482 34879219

[16] Bossonario PA, Ferreira MRL, Andrade RL de P, et al. Risk factors for HIV infection among adolescents and the youth: A systematic review. Rev Lat Am Enfermagem. 2022;30(spe):e3696. 10.1590/1518-8345.6264.3696 PMC9647917 36197391

[17] Chawla N, Sarkar S. Defining “high-risk sexual behavior” in the context of substance use. J Psychosex Health. 2019;1(1):26–31. 10.1177/2631831818822015

[18] Muche AM, Kassa GM, Berhe AK, Fekadu GA. Prevalence and determinants of risky sexual practice in Ethiopia: Systematic review and Meta-analysis. Reprod Health. 2017;14:113. 10.1186/s12978-017-0376-4 28877736 PMC5588747

[19] Azeze GA, Gebeyehu NA, Wassie AY, Mokonnon TM. Factors associated with risky sexual behaviour among secondary and preparatory students in Wolaita Sodo town, Southern Ethiopia; Institution based cross-sectional study. Afr Health Sci. 2021;21(4):1830. 10.4314/AHS.V21I4.41 35283985 PMC8889837

[20] Mbizvo MT, Kasonda K, Muntalima NC, et al. Comprehensive sexuality education linked to sexual and reproductive health services reduces early and unintended pregnancies among in-school adolescent girls in Zambia. BMC Public Health. 2023;23(1):1–13. 10.1186/S12889-023-15023-0/TABLES/4 36797703 PMC9933327

[21] Chavula MP, Zulu JM, Hurtig AK. Factors influencing the integration of comprehensive sexuality education into educational systems in low- and middle-income countries: A systematic review. Reprod Health. 2022;19(1):196. 10.1186/S12978-022-01504-9 PMC9524136 36175901

[22] Zulu JM, Blystad A, Haaland MES, Michelo C, Haukanes H, Moland KM. Why teach sexuality education in school? Teacher discretion in implementing comprehensive sexuality education in rural Zambia. Int J Equity Health. 2019;18(1):1–10. 10.1186/s12939-019-1023-1 31558168 PMC6764121

[23] Chavula MP, Zulu JM, Goicolea I, Hurtig AK. Unlocking policy synergies, challenges and contradictions influencing implementation of the comprehensive sexuality education framework in Zambia: A policy analysis. Health Res Policy Syst. 2023;21(1):1–15. 10.1186/s12961-023-01037-y PMC10500755 37710251

[24] Kirby DB, Laris BA, Rolleri LA. Sex and HIV education programs: Their impact on sexual behaviors of young people throughout the world. J Adolesc Health. 2007;40(3):206–217. 10.1016/J.JADOHEALTH.2006.11.143 17321420

[25] Ministry of Health Z. Zambia population-based hiv impact assessment 2021 [homepage on the Internet]. 2021 [cited 2024 May 20]. Available from: https://www.zamstats.gov.zm/wp-content/uploads/2023/12/ZAMPHIA-2021-Final-Report-December-2023.pdf

[26] Sherinah K, Saasa OM. Determinants of HIV-risk sexual behaviors among Zambian adolescents: The role of gendered power. Child Youth Serv Rev. 2019;106:104484. 10.1016/j.childyouth.2019.104484

[27] Butts SA, Kayukwa A, Langlie J, et al. HIV knowledge and risk among Zambian adolescent and younger adolescent girls: Challenges and solutions. Sex Educ. 2018;18(1):1–13. 10.1080/14681811.2017.1370368 31275062 PMC6606053

[28] Ministry of Education, Science, Vocational Training and Early Education. Comprehensive sexuality education framework. Curriculum Development Centre; 2013 [cited 2024 May 15]. Available from: https://www.comprehensivesexualityeducation.org/wp-content/uploads/CSE-Framework.pdf

[29] Yang X, Yuan S, Zhang R, et al. Risky sexual behaviors and associated factors among college students in Lusaka, Zambia. Arch Sex Behav. 2019;48(7):2117–2123. 10.1007/S10508-019-1442-5 31309429

[30] Mwanza J, Kawonga M, Kumwenda A, Gray GE, Mutale W, Doherty T. Health system response to preventing mother-to-child transmission of HIV policy changes in Zambia: A health system dynamics analysis of primary health care facilities. Glob Health Action. 2022;15(1):2126269. 10.1080/16549716.2022.2126269 36239946 PMC9578454

[31] Kitwe (District, Zambia) – Population statistics, charts, map and location [homepage on the Internet]. [cited 2023 Sept 13]. Available from: https://www.citypopulation.de/en/zambia/wards/admin/0204__kitwe/

[32] Kemigisha E, Bruce K, Ivanova O, et al. Evaluation of a school based comprehensive sexuality education program among very young adolescents in rural Uganda. BMC Public Health. 2019;19(1):1–11. 10.1186/S12889-019-7805-Y/TABLES/5 31660918 PMC6819440

[33] Pengpid S, Peltzer K. Sexual risk behaviour and its correlates among adolescents in Mozambique: Results from a national school survey in 2015. SAHARA J J Soc Asp HIV/AIDS Res Alliance. 2021;18(1):26–32. 10.1080/17290376.2020.1858947 PMC7919870 33641602

[34] Bang-on Thepthien & Celyn. Risky sexual behavior and associated factors among sexually-experienced adolescents in Bangkok, Thailand: Findings from a school web-based survey. Reprod Health. 2022;19(1). 10.1186/S12978-022-01429-3 PMC9148491 35643503

[35] Manu A, Ogum-Alangea D, Azilaku JC, Anaba EA, Torpey K. Risky sexual behaviours and HIV testing among young people in Ghana: Evidence from the 2017/2018 Multiple Indicator Cluster Survey. Manual Reprod Health. 2022;19:125. 10.1186/s12978-022-01439-1 PMC9148450 35643502

[36] Letamo G, Mokgatlhe LL. Predictors of risky sexual behaviour among young people in the era of HIV/AIDS: Evidence from the 2008 Botswana AIDS impact survey III. Afr J Reprod Health [serial online]. 2013 [cited 2024 May 22];17(3):169–181. Available from: https://pubmed.ncbi.nlm.nih.gov/24069779 24069779

[37] James PB, Osborne A, Babawo LS, Bah AJ, Margao EK. The use of condoms and other birth control methods among sexually active school-going adolescents in nine sub-Saharan African countries. BMC Public Health. 2022;22(1):1–11. 10.1186/s12889-022-14855-6 36527019 PMC9756616

[38] Yoon S, Voith LA, Kobulsky JM. Gender differences in pathways from child physical and sexual abuse to adolescent risky sexual behavior among high-risk youth. J Adolesc. 2018;64(1):89–97. 10.1016/J.ADOLESCENCE.2018.02.006 29438874

[39] Lalor K, McElvaney R. Child sexual abuse, links to later sexual exploitation/high-risk sexual behavior, and prevention/treatment programs. Trauma Violence Abuse. 2010;11(4):159–177. 10.1177/1524838010378299 20679329

[40] Shamu S, Shamu P, Zarowsky C, Temmerman M, Shefer T, Abrahams N. Does a history of sexual and physical childhood abuse contribute to HIV infection risk in adulthood? A study among post-natal women in Harare, Zimbabwe. PLoS One. 2019;14(1):e0198866. 10.1371/JOURNAL.PONE.0198866 30608938 PMC6319705

[41] Richter L, Komárek A, Desmond C, et al. Reported physical and sexual abuse in childhood and adult HIV risk behaviour in three African countries: Findings from project accept (HPTN-043). AIDS Behav. 2014;18(2):381. 10.1007/S10461-013-0439-7 23474641 PMC3796176

[42] Haile RT, Kebeta ND, Kassie GM. Prevalence of sexual abuse of male high school students in Addis Ababa, Ethiopia. BMC Int Health Hum Rights. 2013;13(1):1–8. 10.1186/1472-698X-13-24 23680171 PMC3682909

[43] Banwari GH. Adolescent male peer sexual abuse: An issue often neglected. Indian J Psychol Med. 2013;35(4):394. 10.4103/0253-7176.122236 24379502 PMC3868093

[44] Barr AL, Knight L, França-Junior I, Allen E, Naker D, Devries KM. Methods to increase reporting of childhood sexual abuse in surveys: The sensitivity and specificity of face-to-face interviews versus a sealed envelope method in Ugandan primary school children. BMC Int Health Hum Rights. 2017;17(1):1–11. 10.1186/s12914-016-0110-2 28231854 PMC5324203

[45] Choudhry V, Agardh A, Stafström M, Östergren PO. Patterns of alcohol consumption and risky sexual behavior: A cross-sectional study among Ugandan university students. BMC Public Health. 2014;14(1):1–11. 10.1186/1471-2458-14-128 24502331 PMC3933239

[46] Amare T, Yeneabat T, Amare Y. A systematic review and meta-analysis of epidemiology of risky sexual behaviors in college and university students in Ethiopia, 2018. J Environ Public Health. 2019;2019:4852130. 10.1155/2019/4852130 31015844 PMC6446110

[47] Cho HS, Yang Y. Relationship between alcohol consumption and risky sexual behaviors among adolescents and young adults: A meta-analysis. Int J Public Health. 2023;68:1605669. 10.3389/IJPH.2023.1605669 37153699 PMC10154531

[48] Hong SW, Suh YS, Kim DH. The risk factors of sexual behavior among middle school students in South Korea. Int J Sex Health. 2018;30(1):72–80. 10.1080/19317611.2018.1429513

[49] De Vlieg RA, Van Empel E, Montana L, et al. Alcohol consumption and sexual risk behavior in an aging population in rural South Africa. AIDS Behav. 2021;25: 2023–2025. 10.1007/s10461-020-03132-5 33387135 PMC8169519

[50] Lavikainen HM, Lintonen T, Kosunen E. Sexual behavior and drinking style among teenagers: A population-based study in Finland. Health Promot Int. 2009;24(2):108–119. 10.1093/heapro/dap007 19304992

[51] Mitiku Dana L, Mehretie Adinew Y, Molla Sisay M. Transactional sex and HIV risk among adolescent school girls in Ethiopia: Mixed method study. 2019;2019:4523475. 10.1155/2019/4523475 PMC6620836 31346517

[52] Ige OS, Solanke BL. Drivers of transactional sexual relationships among students in a Nigerian University: Implications for elimination of reproductive rights violation. Int J Reprod Contracept Obstet Gynecol. 2021;10(1):55–60. 10.18203/2320-1770.IJRCOG20205754

[53] Mathur S, Pilgrim N, Patel SK, et al. HIV vulnerability among adolescent girls and young women: A multi-country latent class analysis approach. Int J Public Health. 2020;65(4):399–411. 10.1007/S00038-020-01350-1 32270233 PMC7274997

[54] Ene JC, Nnama-Okechukwu CU, Anazonwu NP. Transactional sex at the University of Nigeria, Nsukka campus: Implications for school social work in Nigeria. J Soc Work Dev Soc. 2021;2(1):37–53.

[55] Pichon M, Howard-Merrill L, Wamoyi J, Buller AM, Kyegombe N. A qualitative study exploring parent–daughter approaches for communicating about sex and transactional sex in Central Uganda: Implications for comprehensive sexuality education interventions. J Adolesc. 2022;94(6):880–891. 10.1002/JAD.12071 35797512

[56] Ranganathan M, Heise L, Pettifor A, et al. Transactional sex among young women in rural South Africa: Prevalence, mediators and association with HIV infection. J Int AIDS Soc. 2016;19(1):20749. 10.7448/IAS.19.1.20749 27469061 PMC4965597

[57] De Oliveira T, Kharsany ABM, Gräf T, et al. Transmission networks and risk of HIV infection in KwaZulu-Natal, South Africa: A community-wide phylogenetic study. Lancet HIV. 2017;4(1):e41. 10.1016/S2352-3018(16)30186-2 27914874 PMC5479933

[58] Kyegombe N, Meiksin R, Wamoyi J, Heise L, Stoebenau K, Buller AM. Sexual health of adolescent girls and young women in Central Uganda: Exploring perceived coercive aspects of transactional sex. Sex Reprod Heal Matters. 2020;28(1):1700770. 10.1080/26410397.2019.1700770 PMC7888006 31934824

[59] Krisch M, Averdijk M, Valdebenito S, Eisner M. Sex trade among youth: A global review of the prevalence, contexts and correlates of transactional sex among the general population of youth. Adolesc Res Rev. 2019;4(2):115–134. 10.1007/s40894-019-00107-z

[60] Dunkle KL, Jewkes R, Nduna M, et al. Transactional sex with casual and main partners among young South African men in the rural Eastern Cape: Prevalence, predictors, and associations with gender-based violence. Soc Sci Med. 2007;65(6):1235–1248. 10.1016/J.SOCSCIMED.2007.04.029 17560702 PMC2709788

[61] Hegdahl HK, Musonda P, Svanemyr J, et al. Effects of economic support, comprehensive sexuality education and community dialogue on sexual behaviour: Findings from a cluster-RCT among adolescent girls in rural Zambia. Soc Sci Med. 2022;306:115125. 10.1016/j.socscimed.2022.115125 35724585

[62] Schaefer R, Gregson S, Eaton JW, et al. Age-disparate relationships and HIV incidence in adolescent girls and young women: Evidence from Zimbabwe. AIDS. 2017;31(10):1461–1470. 10.1097/QAD.0000000000001506 28426534 PMC5457819

[63] UNAIDS. Transactional sex and HIV risk: From analysis to action [homepage on the Internet]. 2018. [cited 2024 May 05]. Available from: https://www.unaids.org/sites/default/files/media_asset/transactional-sex-and-hiv-risk_en.pdf

[64] Maughan-Brown B, George G, Beckett S, et al. HIV risk among adolescent girls and young women in age-disparate partnerships: Evidence from KwaZulu-Natal, South Africa. J Acquir Immune Defic Syndr. 2018;78(2):155–162. 10.1097/QAI.0000000000001656 29767637 PMC5968825

[65] George G, Beckett S, Reddy T, et al. Determining HIV risk for adolescent girls and young women (AGYW) in relationships with “Blessers” and age-disparate partners: A cross-sectional survey in four districts in South Africa. BMC Public Health. 2022;22:973. 10.1186/s12889-022-13394-4 35568839 PMC9107706

[66] Moore EW, Berkley-Patton JY, Hawes SM. Religiosity, alcohol use, and sex behaviors among college student-athletes. J Relig Health. 2013;52(3):930–940. 10.1007/s10943-011-9543-z 21979810

[67] Mabaso M, Mlangeni L, Makola L, et al. Emerging themes in epidemiology volume 18 A number: 3 (2021) C this article. Factors associated with age-disparate sexual partnerships among males and females in South Africa: A multinomial analysis of the 2012 national population-based household survey data. Emerg Themes Epidemiol. 2021;18:3. 10.1186/s12982-021-00093-5 33706776 PMC7953539

[68] Ranganathan M, Quinones S, Palermo T, Gilbert U, Kajula L. Transactional sex among adolescent girls and young women enrolled in a cash plus intervention in rural Tanzania: A mixed-methods study. J Int AIDS Soc. 2022;25(12):e26038. 10.1002/JIA2.26038 36451279 PMC9712808

[69] Pei R, Ji-Ke C, Yang S, et al. Risk factors for HIV infection among 15 to 25-year-old rural unmarried Yi adolescents in an ethnic minority region of China. Medicine (United States). 2018;97(36):e12279. 10.1097/MD.0000000000012279 PMC6133621 30200171

[70] Awotidebe A, Phillips J, Lens W. Factors contributing to the risk of HIV infection in rural school-going adolescents. Int J Env Res Public Health. 2014;11(11):11805–11821. 10.3390/ijerph111111805 25405598 PMC4245644

[71] Le Mat MLJ, Miedema EAJ, Amentie SA, Kosar-Altinyelken H. Moulding the teacher: Factors shaping teacher enactment of comprehensive sexuality education policy in Ethiopia. 2019;51(6):862–880. 10.1080/03057925.2019.1682967

[72] Eaton LA, Cain DN, Pope H, Garcia J, Cherry MC. The relationship between pornography use and sexual behaviors among at-risk HIV negative men who have sex with men. Sex Health. 2012;9(2):166–170. 10.1071/SH10092 22498161 PMC3560402

[73] Mattebo M, Tydén T, Häggström-Nordin E, Nilsson KW, Larsson M. Pornography consumption, sexual experiences, lifestyles, and self-rated health among male adolescents in Sweden. J Dev Behav Pediatr. 2013;34(7):460–468. 10.1097/DBP.0B013E31829C44A2 23899659

[74] Harkness EL, Mullan BM, Blaszczynski PA. Association between pornography use and sexual risk behaviors in adult consumers: A systematic review. Cyberpsychol Behav Soc Netw. 2015;18(2):59–71. 10.1089/cyber.2014.0343 25587721

[75] Rasmussen EE, Rhodes N, Ortiz RR, White SR. The relation between norm accessibility, pornography use, and parental mediation among emerging adults. Media Psychol. 2026;19(3):431–454. 10.1080/15213269.2015.1054944

